# Strain ultrasonic elastography imaging features of locally advanced breast cancer: association with response to neoadjuvant chemotherapy and recurrence-free survival

**DOI:** 10.1186/s12880-023-01168-2

**Published:** 2023-12-21

**Authors:** Caifeng Wan, Liheng Zhou, Ye Jin, Fenghua Li, Lin Wang, Wenjin Yin, Yaohui Wang, Hongli Li, Lixin Jiang, Jinsong Lu

**Affiliations:** 1 Department of Breast Surgery, Renji Hospital, Shanghai Jiao Tong University School of Medicine, Pujian Rd, Shanghai, 200127 China; 2 Department of Ultrasound, Renji Hospital, Shanghai Jiao Tong University School of Medicine, Pujian Rd, Shanghai, 200127 China

**Keywords:** Neoadjuvant chemotherapy, Locally advanced breast cancer, Strain elastography, Strain ratio, Recurrence-free survival

## Abstract

**Background:**

Due to the highly heterogeneity of the breast cancer, it would be desirable to obtain a non-invasive method to early predict the treatment response and survival outcome of the locally advanced breast cancer (LABC) patients undergoing neoadjuvant chemotherapy (NAC). This study aimed at investigating whether strain elastography (SE) can early predict the pathologic complete response (pCR) and recurrence-free survival (RFS) in LABC patients receiving NAC.

**Methods:**

In this single-center retrospective study, 122 consecutive women with LABC who underwent SE examination pre-NAC and after one and two cycles of NAC enrolled in the SHPD001(NCT02199418) and SHPD002 (NCT02221999) trials between January 2014 and August 2017 were included. The SE parameters (Elasticity score, ES; Strain ratio, SR; Hardness percentage, HP, and Area ratio, AR) before and during NAC were assessed. The relative changes in SE parameters after one and two cycles of NAC were describe as ΔA_1_ and ΔA_2_, respectively. Logistic regression analysis and Cox proportional hazards model were used to identify independent variables associated with pCR and RFS.

**Results:**

Forty-nine (40.2%) of the 122 patients experienced pCR. After 2 cycles of NAC, SR_2_ (odds ratio [OR], 1.502; *P* = 0.003) and ΔSR_2_ (OR, 0.013; *P* = 0.015) were independently associated with pCR, and the area under the receiver operating characteristic curve for the combination of them to predict pCR was 0.855 (95%CI: 0.779, 0.912). Eighteen (14.8%) recurrences developed at a median follow-up of 60.7 months. A higher clinical T stage (hazard ratio [HR] = 4.165; *P* = 0.005.), a higher SR (HR = 1.114; *P* = 0.002.) and AR (HR = 1.064; *P* <  0.001.) values at pre-NAC SE imaging were independently associated with poorer RFS.

**Conclusion:**

SE imaging features have the potential to early predict pCR and RFS in LABC patients undergoing NAC, and then may offer valuable predictive information to guide personalized treatment.

## Introduction

Over the last decades, neoadjuvant chemotherapy (NAC) followed by surgery has become the standard treatment for patients with locally advanced breast cancer (LABC) [1]. Patients who failed to achieve pathologic complete response (pCR) were proved to be the strongest independent risk factor for recurrence [2]. Due to the high heterogeneity of breast cancer, the response to chemotherapy is different, and only a minority of patients achieve pCR [3–8]. Therefore, it would be desirable to obtain a non-invasive method to early predict the treatment response and survival outcome of the LABC patients undergoing NAC.

Stiffness is one of the most important biomechanical properties of the breast cancers [9]. Ultrasonic elastography which can measure tissue stiffness non-invasively was considered to be a unique technique to provide improved detection and characterization of breast tumors [10, 11], and was reported to be a powerful tool to predict response early during the course of chemotherapy [12, 13]. Collagen-rich extracellular matrix (ECM) remodeling is one of the key features involved in tumor development, progression and drug resistance [14]. The stiffness of the tumoral stroma can be enhanced through increased secretion of ECM proteins as well as the thickening and reorganization of the collagen fibrils [15]. Tumor stiffness has been shown to be significantly correlated with tumor growth and aggression, and high mean stiffness values of breast cancers were reported to be significantly correlated with large invasive size and high histologic grade [9]. Higher tumor stiffness assessed by shear-wave elastography (SWE) was associated with worse disease-free survival in patients with early-stage invasive breast cancer [16]. Ultrasonic elastography may be an ideal investigative modality for early prediction of response to chemotherapy and recurrence-free survival (RFS) in breast cancer patients without exposing the patients to any risk of radiation and external contrast agent. The two most frequently used ultrasound elastography techniques for the examination of breast tumors are strain elastography (SE) and SWE. No significant difference was found between them in differentiating diagnosis of breast tumors and in early predicting of NAC response [17, 18].

This study was, therefore, an endeavor to further evaluate whether SE parameters and their changes at different time-points during NAC were associated with pCR. As far as we know, no study investigated the efficacy of pre-NAC SE imaging features for predicting the RFS of LABC patients after NAC. Therefore, the purpose of our study was to determine the optimal parameters of SE and the ideal time-point for early prediction of pCR to NAC, and the relationship between pre-NAC SE imaging features and RFS in LABC patients receiving NAC was also investigated.

## Materials and methods

### Study population

**Fig. 1:**
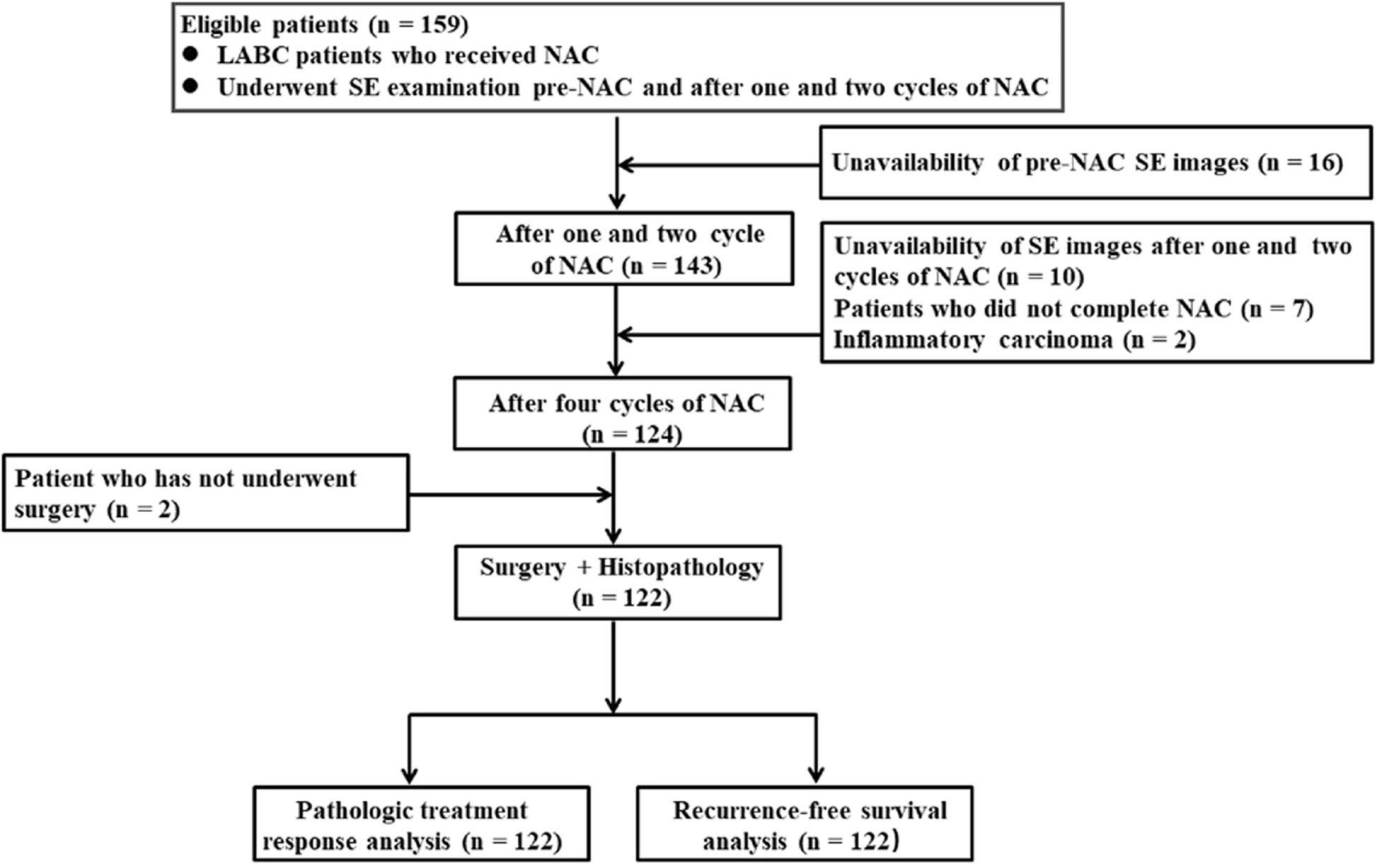
A flow diagram of patient enrollment process. LABC = Locally advanced breast cancer, NAC = Neoadjuvant chemotherapy, SE = Strain elastography

Our institutional review board approved this retrospective study. Consecutive women aged 18 to 70 years with LABC who underwent SE examination pre-NAC and after one and 2 cycles of NAC between January 2014 and August 2017 were considered for inclusion. We excluded patients who did not have evaluable SE images pre-NAC and after one and 2 cycles of NAC (*n* = 16), those who had inflammatory carcinoma (*n* = 2), those who did not finished full cycles of NAC (*n* = 7) and those who did not undergo surgery after NAC (*n* = 2). Accordingly, 122 women (mean age, 51.1 years; range, 25–70 years) comprised the study group for pathologic treatment response and RFS analysis (Fig. 1).

All patients in this study were from two separately registered prospective NAC clinical trials, SHPD001(NCT02199418) and SHPD002 (NCT02221999). The protocols of the studies were published previously [19]. All patients received paclitaxel-cisplatin-based NAC. In short, cisplatin (25 mg/m^2^) on day 1,8, and 15 every 28 days combined with paclitaxel (80 mg/m^2^) on day 1, 8, 15, and 22 for 4 cycles. Concomitant trastuzumab was recommended for HER2-positive patients at a loading dose of 4 mg/kg followed by a maintenance dose of 2 mg/kg, weekly, for 16 weeks. Hormone receptor-positive breast cancer patients were randomized to chemotherapy combined with endocrine therapy on the basis of their menstrual status or chemotherapy alone in SHPD002. In SHPD002, premenopausal patients with triple negative breast cancer were randomized to chemotherapy with or without ovarian function suppression.

### Ultrasonic elastography examination and analysis

All patients underwent SE examination pre-NAC and after one and 2 cycles of NAC. Conventional and SE US were obtained with MyLab Twice (Esaote, Genoa, Italy) equipped with a 4–13-MHz LA523 linear transducer. The selected plane for SE examination should include the lesion and its surrounding normal tissue (generally from the subcutaneous fat to the pectoral muscle). During SE examination, the radiologists vertically compressed the skin above the targeted lesion with the probe under light and steady pressure. The real-time elaXto-spring tool which displayed in the lower-left corner of the screen helps radiologists obtained ideal SE images by adjusting the pressure and frequency of compression. Patients were instructed to suspend respiration for 3–5 s during the data acquisition to reduce motion artifacts. The stiffness of the tissue was displayed in a color-coded mode, with blue, green and red colors representing hard, intermediate and soft tissue, respectively. SE examination was performed by two radiologists (Y.J and L.W. with 8 and 9 years of experience in breast SE examination, respectively).

SE images were reviewed by consensus of two radiologists (C.F.W and H.F. with 10 and 11 years of experience in interpreting breast SE images, respectively) blinded to clinical and pathologic data. The elastic score is based on 5-point scoring system [20]. A region of interest (ROI) was defined manually around the whole lesion, and the areas of necrosis and calcifications were avoided. The corresponding ROI in adjacent normal fatty was also manually drawn as a control. In the semi-quantitative evaluation of the elastographic images, the strain ratio (SR) was defined as the fat-to-mass SR which represents the relative stiffness of the tumor to the fat tissue. The hardness percentage (HP) could offer percentage value for the hardest part of the tumor. The area ratio (AR) was calculated as the area of the tumor measured in the elastographic image divided by the area of the tumor measured in the conventional US. ES, SR, HP and AR measurements from three different sections were averaged, and the mean value was used for subsequent analysis. The relative changes in tumor diameter and SE parameters after one and 2 cycles of NAC were described as ΔA_1_ and ΔA_2_, and were calculated by the formula: ΔA = ([value before NAC-value after NAC] / value before NAC) × 100%.

### Clinical-pathologic analysis

Baseline information of the patients were collected including patients’ age, body mass index (BMI), menopausal status, tumor size at US, clinical tumor T stage, clinical nodal N stage, estrogen receptor (ER), progesterone receptor (PR), human epidermal growth factor receptor 2 (HER-2), Ki-67 proliferative index status, adjuvant endocrine therapy and adjuvant Herceptin. Pathologic response was assessed by two board-certified breast pathologists both with more than 15 years of experience. ER and PR positivity were defined as ≥1% tumor cells have nuclear staining with any intensity. HER2 positivity was defined as immuno-histochemistry 3+ or amplified for fluorescence in-situ hybridization [21]. The cutoff value for Ki-67 high-level expression was 30%. Molecular subtypes were categorized according to the St. Gallen Consensus [22]: luminal A like (ER positive and/or PR positive, HER2 negative, and Ki-67 < 15%), luminal B like (ER or PR positive; HER2 negative and Ki67 ⩾ 15%, or HER2 positive and any Ki67 index), HER2-enriched (ER and PR negative, and HER2 positive), and triple-negative (ER, PR and HER2 negative). In this study, pCR was defined as the absence of residual invasive cancer in breast with the absence of axillary lymph node involvement.

### Statistical analysis

Differences in categorical variables were analyzed with either the Pearson *x*^*2*^ test or Fisher’s exact test. For the continuous variables, Student’s t-test or Mann-Whitney U test was performed. Univariate and multivariate logistic regression analyses were performed to identify independent variables associated with pCR. Receiver operating characteristic (ROC) curves were analyzed and the areas under the ROC curves (AUC) were calculated.

RFS was defined as the time from surgery to the date of the first recurrence. Recurrence was defined as local-regional recurrence (ipsilateral breast or chest wall and/or axillary, infraclavicular, or supraclavicular lymph nodes) and distant metastasis. In patients who did not develop recurrence was defined as the interval between the date of surgery and the last follow-up. Patients who did not develop recurrence at the last follow-up were treated as censored observations. Cox proportional hazards model was used for univariate and multivariate analyses to determine the association of the clinical-pathologic variables and SE parameters before NAC with RFS. Variables with P<0.1 on the univariate analysis were included in the multivariate Cox regression analysis through the forward stepwise selection method. Survival curves were estimated using Kaplan-Meier analysis, and survival rates were compared by log-rank test. For Kaplan-Meier survival curves, the optimal cutoff values defined by the ROC curves were used to dichotomize the SE parameters into two groups.

All analyses were performed with IBM SPSS Statistics 22 (Armonk, NY, USA) except for the ROC curves analysis, which were performed by MedCalc (version 11.2.1.0; MedCalc Software, Mariakerke, Belgium). *P* < .05 was considered to indicate a statistically significant difference.

## Results

### Patient characteristics

Baseline clinicopathological characteristics of all patients are summarized in Table 1. The median tumor size measured at US was 4.2 cm (95% CI: 39.29, 45.26), range from 1.6 to 10 cm. Of the 122 breast cancers, 113 were invasive ductal carcinoma, four were invasive micropapillary carcinoma, two were invasive lobular carcinoma, two were mucinous carcinoma and one was a tubular carcinoma.

**Table 1:** Baseline characteristics of the study patients

Characteristic	pCR	Non-pCR	*P* value	Recurrence Group	Nonrecurrence Group	Total number	*P* Value
Number of lesions^b^	49 (40.16)	73 (59.83)		18 (14.75)	104 (85.24)	122	
Age (y) ^a^	51 (26–70)	51 (25–70)	0.135	50 (25–67)	52 (26–70)	ZZ	0.61
Tumor diameter (cm) ^a^	4.0 (1.4–8.4)	4.4 (2.2–10.0)	0.14	4.8 (2.1–10.0)	4.1 (1.4–8.7)		0.097
Menopausal status^b^			0.395				0.508
Premenopausal	23 (46.94)	40 (54.79)		8 (44.44)	55 (52.88)	63 (51.22)	
Postmenmopausal	26 (53.06)	33 (45.21)		10 (55.56)	49 (47.12)	59 (48.36)	
Clinical T stage^b^			0.039				< 0.001
T_1 ~ 3_	40 (81.63)	47 (64.38)		6 (33.33)	81 (77.88)	87 (71.31)	
T_4_	9 (18.37)	26 (35.62)		12 (66.67)	23 (22.12)	35 (28.69)	
Clinical N stage^b^			0.33				0.01
N_0 ~ 2_	44 (89.79)	61 (83.56)		12 (66.67)	93 (89.42)	105(86.07)	
N_3_	5 (10.21)	12 (16.44)		6 (33.33)	11 (10.48)	17 (13.93)	
ER status^b^			0.005				0.803
ER negative	19 (38.78)	12 (16.44)		5 (27.78)	26 (25)	31 (25.41)	
ER positive	30 (61.22)	61 (83.56)		13 (72.22)	78 (75)	91 (74.59)	
PR status^b^			0.02				0.641
PR negative	17 (34.69)	12 (16.44)		3 (16.67)	26 (25)	29 (23.77)	
PR positive	32 (65.31)	61 (83.56)		15 (83.33)	78 (75)	93 (76.23)	
HER2 status^b^			0.001				0.312
HER2 negative	18 (36.73)	50 (68.49)		12 (66.67)	56 (53.85)	68 (55.74)	
HER2 positive	31 (63.27)	23 (31.51)		6 (33.33)	48 (46.15)	54 (44.26)	
Ki-67 index^b^			0.064				0.905
≥30	43 (87.76)	54 (73.97)		15 (83.33)	82 (78.85)	97 (79.51)	
<30	6 (12.24)	19 (26.03)		3 (16.67)	22 (21.15)	25 (20.49)	
Molecular subtype^b^			0.001				0.358
Luminal A-like	0 (0)	10 (13.70)		3 (16.67)	7 (6.73)	10 (8.20)	
Luminal B-like (HER2 positive)	22 (44.90)	19 (26.03)		4 (22.22)	37 (35.58)	41 (33.33)	
Luminal B-like (HER2 negative)	14 (28.57)	36 (49.31)		9 (50)	41 (39.42)	50 (40.98)	
Triple-negative	4 (8.16)	4 (5.48)		0 (0)	8 (7.70)	8 (6.56)	
HER2-enriched	9 (18.37)	4 (5.48)		2 (11.11)	11 (10.58)	13 (10.67)	
BMI^b^			0.675				0.55
<25	34 (69.39)	48 (65.75)		11 (61.11)	71 (68.27)	82 (67.21)	
≥25	15 (30.61)	25 (34.24)		7 (38.89)	33 (31.73)	40 (32.79)	
SE parameters before NAC							
ES	4.43 ± 0.74	4.41 ± 0.57	0.54	4.44 ± 0.62	4.41 ± 0.65		0.907
SR	11.86 ± 4.57	13.10 ± 5.66	0.4	18.44 ± 7.32	11.60 ± 4.09		< 0.001
HP	98.86 ± 2.87	99.43 ± 0.90	0.97	99.91 ± 0.19	99.01 ± 2.10		0.004
AR	112.73 ± 14.17	112.78 ± 12.04	0.985	126.78 ± 16.13	110.34 ± 10.57		< 0.001

Data are number of patients and data in parentheses are percentages. *pCR* Pathologic complete response, *ER* Estrogen receptor, *PR* Progesterone receptor, *HER2* Human epidermal growth factor receptor 2, Luminal A-like (ER-positive and/or PR-positive, HER2-negative, and Ki-67 expression < 14%), Luminal B-like/HER2-positive (ER-positive and/or PR-positive; HER2-positive and Ki-67 expression ⩾ 14%), Luminal B-like/HER2-negative (ER-positive and/or PR-positive; HER2-negative and Ki-67 expression ⩾ 14%), HER2-enriched (ER and PR-negative, and HER2-positive), and Triple-negative (ER-negative, PR-negative, and HER2-negative). *BMI* Body mass index, *SE* Strain elastography, *NAC* Neoadjuvant chemotherapy, *ES* Elasticity score, *SR* Strain ratio, *HP* Hardness percentage, *AR* Area ratio

^a^Data are medians, with ranges in parentheses

^b^Data in the parentheses are percentage

### Pathologic treatment response

Overall, 49 (40.2%) patients achieved pCR after NAC. Sixty-nine patients showed partial response (at least a 30% decrease in the sum of diameters of target lesions, taking as reference the baseline sum diameters) and 4 patients showed stable diseases (Neither sufficient shrinkage to qualify for PR nor sufficient increase to qualify for PD). Patients were more easily to achieve pCR with ER and PR receptor negative (*P* = .005 and .02, respectively), and HER2 receptor positive (*P* = .001). Compared with the nonpCR group, the pCR group had more cases of triple-negative and HER2-enriched breast cancer (*P* = .001). No evidence for a significant difference was found in pre-NAC SE imaging features between pCR and nonpCR groups (Table 1).

### Associations of SE imaging features with PCR

**Fig. 2:**
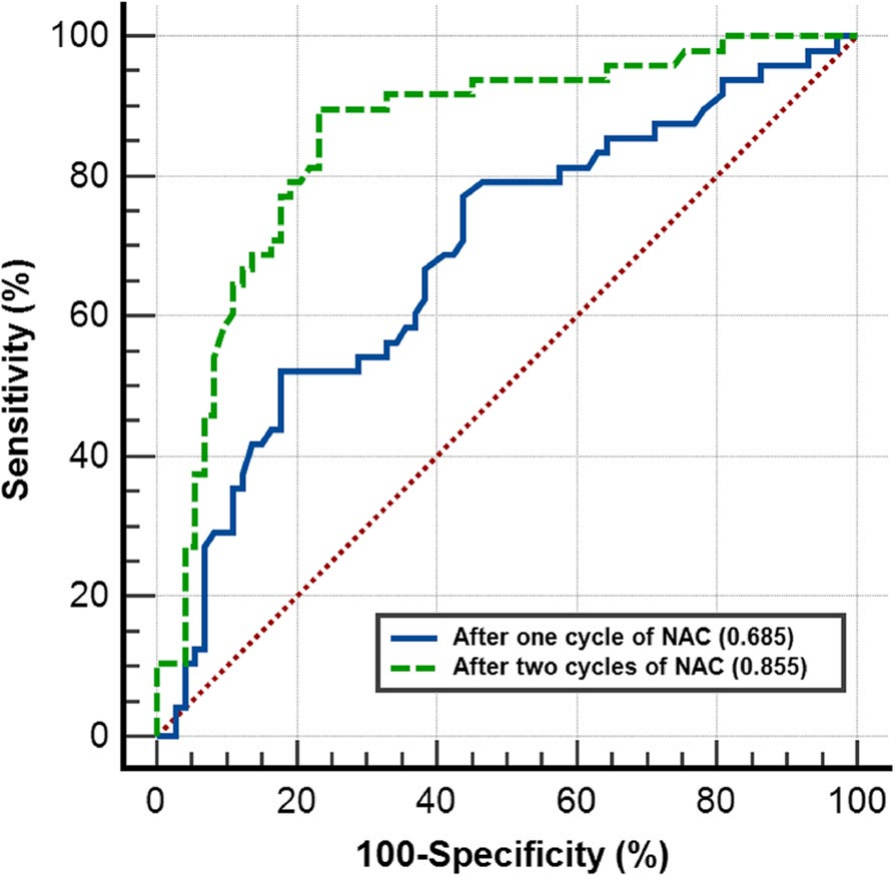
Receiver operating characteristic curves (ROC) of different cycles of NAC. After one cycle of NAC, the area under the ROC (AUC) for strain ratio (SR) to predict pCR was 0.685 (Az1, 95% CI: 0.594, 0.766) (blue solid line). After two cycles of NAC, AUC for the combination of SR_2_ and change in SR_2_ to predict pCR was 0.855 (Az2, 95% CI: 0.779, 0.912) (green dashed line). The value of Az2 was significantly higher than that of Az1 (*P* < .001)

After 1 cycle of NAC, univariate analysis indicated that the ES_1_ (*P* = .001), SR_1_ (*P* = .001), HP_1_ (*P* = .019), ΔSR_1_ (*P* = .002) and ΔHP_1_ (*P* = .021) measurements showed a significant difference between the pCR and nonpCR groups (Table 2). The mean tumor diameter was significantly larger in patients with nonpCR than that in those with pCR (32.57 ± 13.93 vs 27.12 ± 11.10 mm*, P* = .03). Multivariate analysis indicated that SR_1_ (odds ratio [OR], 1.236; 95% CI: 1.093, 1.397; *P* = .001) was independently associated with pCR, and the AUC for SR_1_ (Az1) to predict pCR was 0.685 (95% CI: 0.594, 0.766; sensitivity, 82.2%; specificity, 53.1%) (Fig. 2, blue solid line).

**Table 2:** Univariable and multivariable analyses of pCR in relation to SE parameters after one and two cycles of NAC

Characteristic	pCR	Non-pCR	Univariable Analysis		Multivariable Analysis	
			Odds Ratio	*P* value	Odds Ratio	*P* value
After one cycle of NAC						
ES_1_	3.63 ± 0.67	4.10 ± 0.69	2.707 (1.512, 4.845)	0.001		
SR_1_	6.72 ± 3.29	8.98 ± 3.69	1.209 (1.076, 1.360)	0.001	1.236 (1.093, 1.397)	0.001
HP_1_	95.33 ± 5.94	97.67 ± 3.73	1.126 (1.020, 1.243)	0.019		
AR_1_	106.53 ± 8.40	107.20 ± 8.75	1.009 (0.967, 1.053)	0.67		
△SR_1_	0.41 ± 0.20	0.29 ± 0.19	0.041 (0.006, 0.296)	0.002		
△HP_1_	0.04 ± 0.04	0.02 ± 0.04	0 (0, 0.153)	0.021		
△AR_1_	0.05 ± 0.07	0.04 ± 0.07	0.437 (0.003, 73.341)	0.751		
Diameter_1_	27.12 ± 11.10	32.57 ± 13.93	1.036 (1.004, 1.070)	0.028		
△Diameter_1_	0.30 ± 0.14	0.26 ± 0.14	0.17 (0.013, 2.309)	0.183		
After two cycles of NAC						
ES_2_	2.98 ± 0.60	3.77 ± 0.74	5.652 (2.769, 11.540)	<0.001		
SR_2_	3.67 ± 1.89	6.85 ± 2.71	1.867 (1.475, 2.361)	<0.001	1.502 (1.147, 1.967)	0.003
HP_2_	86.86 ± 9.86	95.85 ± 6.64	1.16 (1.088, 1.236)	<0.001		
AR_2_	100.56 ± 4.79	104.41 ± 13.63	1.041 (0.992, 1.093)	0.101		
△SR_2_	0.67 ± 0.14	0.44 ± 0.20	0.001 (0, 0.009)	<0.001	0.013 (0, 0.429)	0.015
△HP_2_	0.12 ± 0.09	0.04 ± 0.07	0 (0, 0)	<0.001		
△AR_2_	0.11 ± 0.17	0.07 ± 0.13	0.1 (0.006, 1.745)	0.115		
Diameter_2_	20.0 ± 9.78	26.82 ± 12.69	1.06 (1.019, 1.103)	0.004		
△Diameter_2_	0.48 ± 0.20	0.40 ± 0.15	0.06 (0.006, 0.577)	0.015		

Data are means ± standard deviations. *pCR* Pathologic complete response, *NAC* Neoadjuvant chemotherapy, *ES* Elasticity score, *SR* Strain ratio, *HP* Hardness percentage, *AR* Area ratio. The relative changes in diameter and SE parameters after one and two cycles of NAC were describe as ΔA_1_ and ΔA_2_, and were calculated by the formula: ΔA = ([value before NAC-value after NAC] / value before NAC) × 100%

Univariate analysis indicated that the mean value of all SE parameters decreased continuously following NAC. Values of ES_2_, SR_2_, HP_2_, ΔSR_2_ and ΔHP_2_ showed a significant difference between the two groups after 2 cycles of NAC (all *P*<.001) (Table 2). The diameter_2_ was significantly larger in patients with nonpCR than that in those with pCR (*P* = .004). Multivariate analysis indicated that SR_2_ (OR, 1.502; 95% CI: 1.147, 1.967; *P* = .003) and ΔSR_2_ (OR, 0.013; 95% CI: 0, 0.429; *P* = .015) were independent predictors of pCR, and the AUC for the combination of them (Az2) to predict pCR was 0.855 (95% CI: 0.779, 0.912; sensitivity, 76.7%; specificity, 89.6%) (Fig. 2, green dashed line). The value of Az2 was significantly higher than that of Az1 (*P* < .001). This suggests that after 2 cycles of NAC may be the ideal time-point to early predict pCR to NAC for breast cancer patients by using SE.

### Recurrence outcome

Of the 122 patients, 18 (14.75%) recurred after a median follow-up of 60.7 months (range, 10–91 months). The sites of metastases include: bones (*n* = 5, 27.78%), brain (*n* = 3, 16.67%), lung (*n* = 4, 22.22%), contralateral axillary lymph node (*n* = 2, 11.11%), liver (*n* = 3,16.67%) and both bones and lung (*n* = 1, 5.56%). With regard to the clinical-pathologic features, higher clinical T (*P*<.001) and N stage (*P* = .01) were more frequently observed in the recurrence group compared with the nonrecurrence group. The SR (*P*<.001), HP (*P* =. 004) and AR (*P*<.001) values of the recurrence group were significantly higher than that in nonrecurrence group at pre-NAC SE (Table 1).

### Factors associated with RFS: univariable and multivariable analyses

**Fig. 3:**
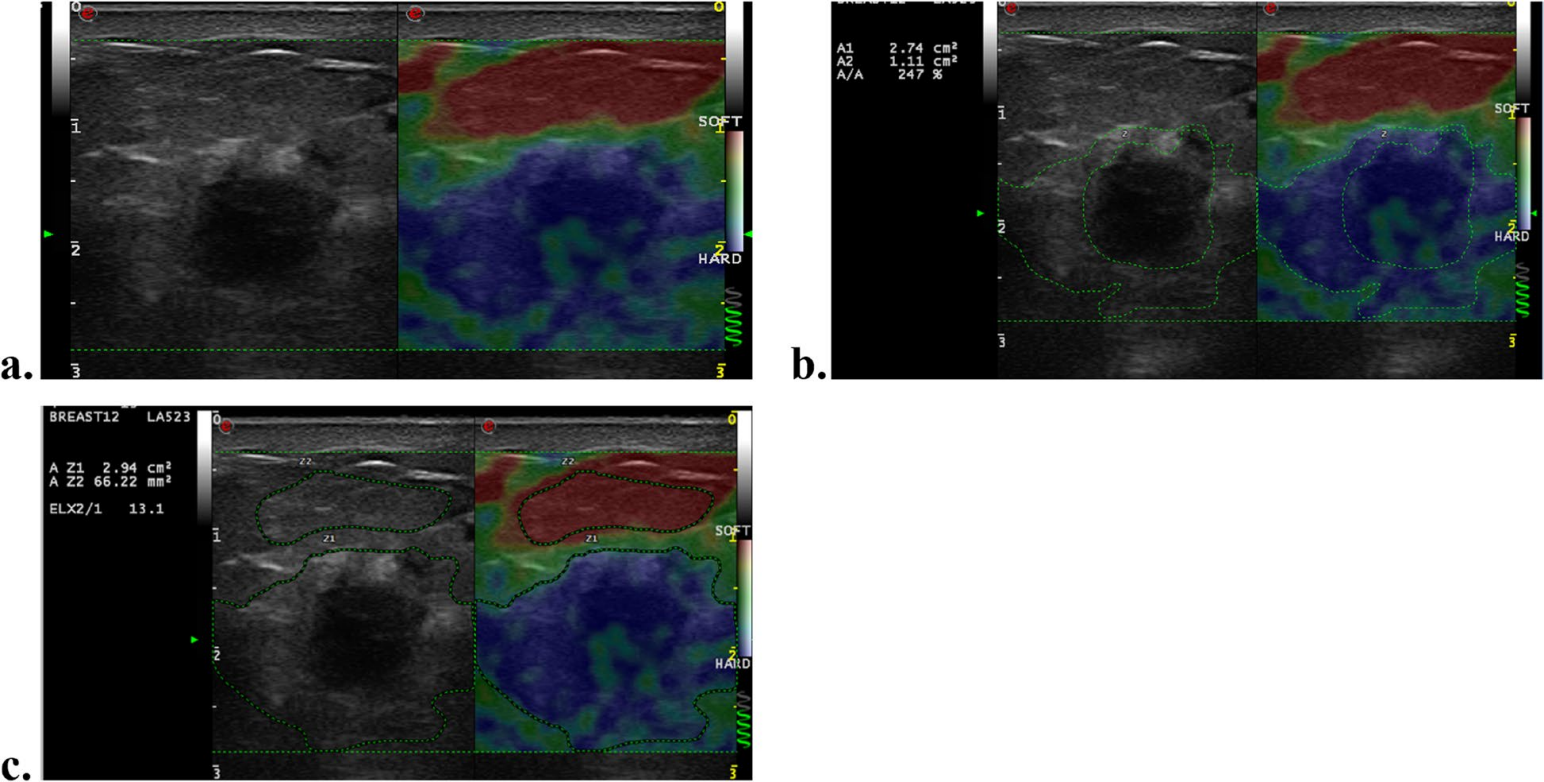
A 56-year-old woman with 3.0 cm luminal B-like breast cancer of the right breast following neoadjuvant chemotherapy (NAC). **a** Grey scale US image shows an irregular hypoechoic mass with indistinct margin. Strain elastography image demonstrates that the lesion was scored 4 before NAC. **b** The area ratio of this lesion before NAC are 247% **c** The strain ratio of this lesion before NAC are 13.1. After surgery, pathological analysis showed only a few scattered tumor cells remained in the breast. This patient was found to have brain metastasis after a follow-up of 24 months

**Fig. 4:**
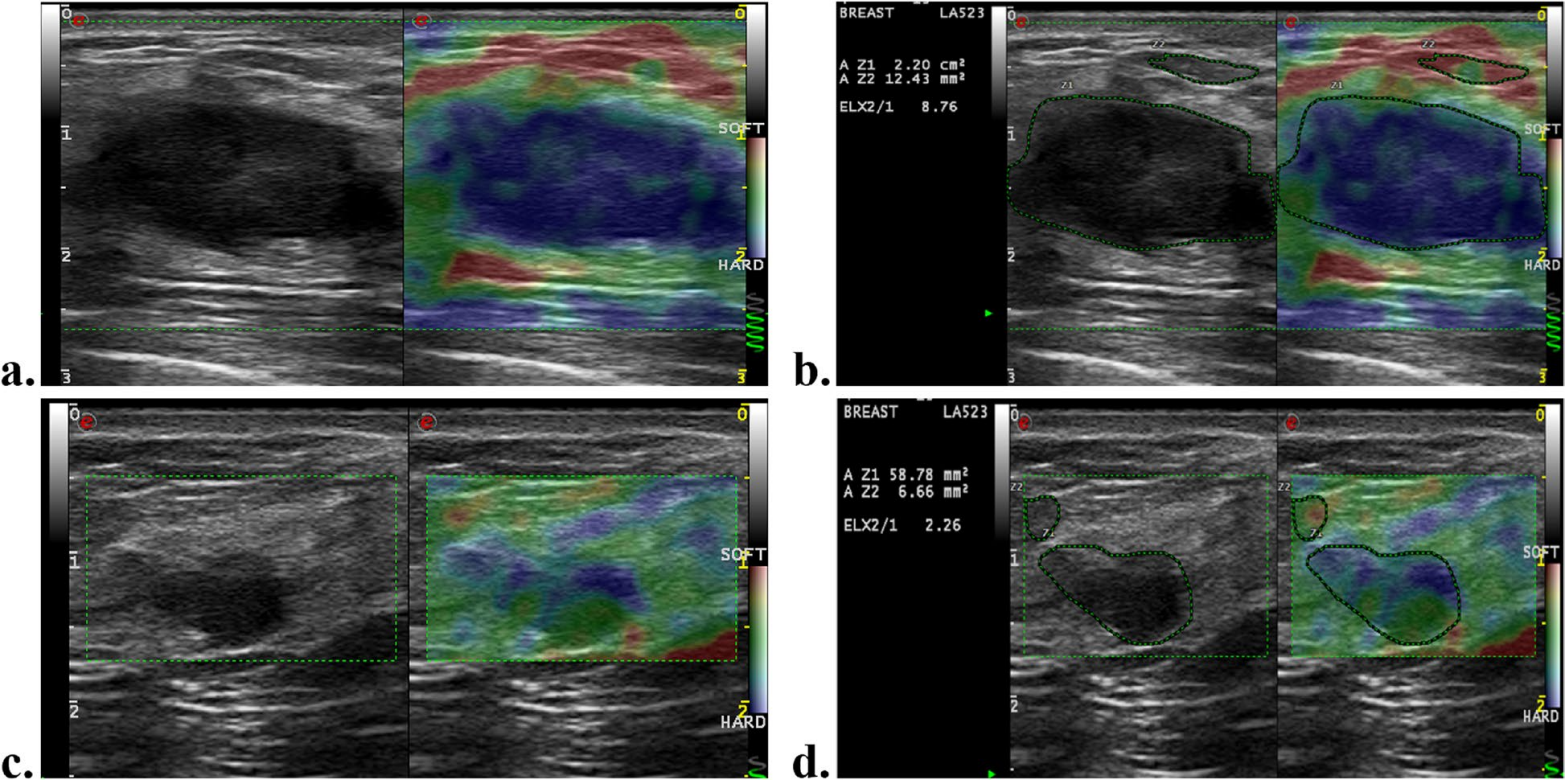
A 45-year-old woman with 3.2 cm triple-negative breast cancer of the left breast following neoadjuvant chemotherapy (NAC). **a** Greyscale US image shows an irregular hypoechoic mass with indistinct margin. Strain elastography image demonstrates that the lesion was scored 4 before NAC. **b** The area ratio of this lesion before NAC are 107%. The strain ratio of this lesion before NAC are 8.76. **c** Strain elastography image demonstrates that the lesion was scored 3 after 2 cycles of NAC. **d** The strain ratio of this lesion after two cycles of NAC are 2.26. After surgery, pathological analysis found no residual cancer in the breast and sampled axillary lymph node. During 71 months of follow-up, there was no evidence of recurrence

Among the clinical-pathologic variables, higher clinical T (hazard ratio [HR] = 5.67; 95% CI: 2.13, 15.11; *P* = .001) and N stage (HR = 3.21; 95% CI: 1.20, 8.57; *P* = .02) were associated with worse RFS. Regarding pre-NAC SE parameters, patients with tumors with higher SR (HR = 1.14; 95% CI:1.01, 1.20; *P*<.001) and AR (HR = 1.09; 95% CI: 1.05, 1.12; *P*<.001) values exhibited worse RFS outcomes. According to multivariate Cox regression analysis, a higher clinical T stage (HR = 4.1; 957% CI: 1.53, 11.32; *P* = .005), higher SR (HR = 1.11; 95% CI:1.04, 1.19; *P* = .002) and AR (HR = 1.06; 95% CI:1.03, 1.09; *P*<.001) values at pre-NAC SE imaging were independently associated with poorer RFS (Table 3). Representative US images of pCR and nonpCR lesions and recurrence and nonrecurrence lesions are given in Figs. 3 and 4.

**Table 3:** Univariable and multivariable cox proportional hazards analyses of variables associated with recurrence–free survival

Characteristic	Univariable Analysis		Multivariable Analysis	
	Hazard Ratio	*P* Value	Hazard Ratio	*P* Value
Age (y)	0.987 (0.948, 1.028)	0.54		
Tumor diameter (mm)	1.021 (0.996, 1.047)	0.102		
Menopausal status		0.539		
Premenopausal	0.747 (0.295, 1.894)			
Postmenmopausal	Ref			
Clinical T stage		0.001		0.005
T1 ~ 3	Ref		Ref	
T4	5.668 (2.126, 15.110)		4.165 (1.532, 11.324)	
Clinical N stage		0.02		
N0 ~ 2	Ref			
N3	3.207 (1.200, 8.569)			
ER status		0.75		
ER negative	0.846 (0.301, 2.373)			
ER positive	Ref			
PR status		0.57		
PR negative	1.433 (0.415, 4.955)			
PR positive	Ref			
HER2 status		0.366		
HER2 negative	0.636 (0.239, 1.695)			
HER2 positive	Ref			
Ki-67 index		0.738		
≥30	1.236 (0.357, 4.274)			
<30	Ref			
Molecular subtype		0.603		
Luminal type	Ref			
Triple negative and HER2-enriched	1.478 (0.340, 6.249)			
BMI		0.553		
<25%	Ref			
≥25%	1.333 (0.516, 3.444)			
SE parameters before NAC				
ES	1.065 (0.511, 2.217)	0.867		
SR	1.138 (1.077, 1.202)	<0.001	1.114 (1.042, 1.191)	0.002
HP	8.508 (0.953, 75.99)	0.055		
AR	1.086 (1.052, 1.120)	<0.001	1.064 (1.032, 1.097)	<0.001

*ER* Estrogen receptor, *PR* Progesterone receptor, *HER2* Human epidermal growth factor receptor 2, Luminal A-like (ER-positive and/or PR-positive, HER2-negative, and Ki-67 expression < 14%), Luminal B-like/HER2-positive (ER-positive and/or PR-positive; HER2-positive and Ki-67 expression ⩾ 14%), Luminal B-like/HER2-negative (ER-positive and/or PR-positive; HER2-negative and Ki-67 expression ⩾ 14%), HER2-enriched (ER and PR-negative, and HER2-positive), and Triple-negative (ER-negative, PR-negative, and HER2-negative). *BMI* Body mass index, *NAC* Neoadjuvant chemotherapy, *SE* Strain elastography, *ES* Elasticity score, *SR* Strain ratio, *HP* Hardness percentage, *AR* Area ratio

**Fig. 5:**
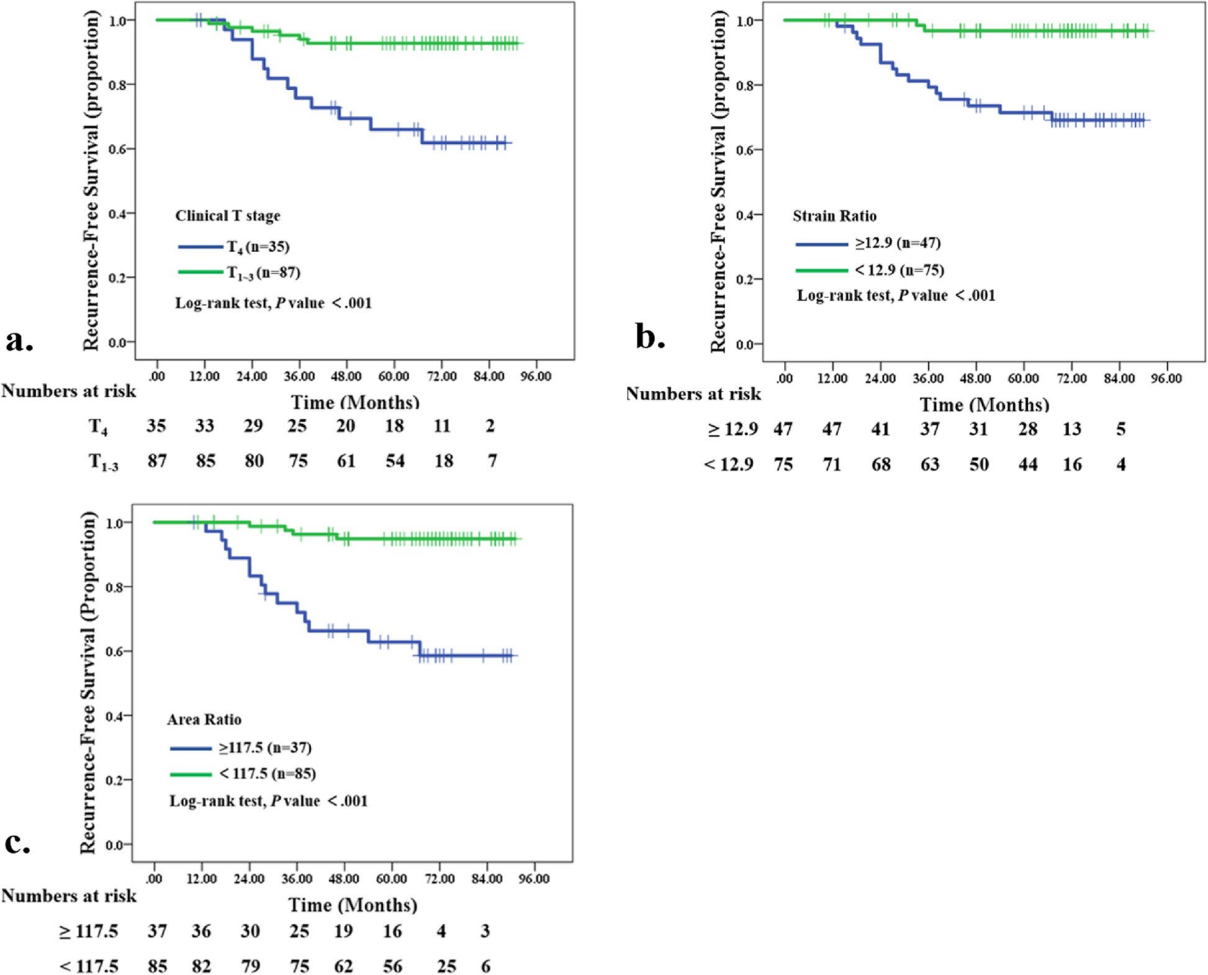
Kaplan-Meier curves show recurrence-free survival (RFS) rates in 122 patients with locally advanced breast cancer receiving neoadjuvant chemotherapy (NAC). **a** Graph shows RFS according to clinical T stage. Blue line = patients with tumors with higher clinical T stage (T_4_) (*n* = 35), green line = patients with tumors with lower clinical T stage (T_1–3_) (*n* = 87) (*P* < .001). **b** Graph shows RFS according to strain ratio (SR) on pre-NAC SE images. Blue line = patients with tumors with higher SR (≥ 12.9) (*n* = 47), green line = patients with tumors with lower SR (<12.9) (*n* = 75) (*P* < .001). **c** Graph shows RFS according to area ratio (AR) on pr-NAC SE images. Blue line = patients with tumors with higher AR (≥ 117.5) (*n* = 37), green line = patients with tumors with lower AR (<117.5) (*n* = 85) (*P* < .001)

At ROC curves analyses, the optimal cutoff values for SR and AR to assess an association with RFS was 12.5 (AUC, 0.805; 95% CI: 0.69, 0.92; sensitivity, 88.9%; specificity, 64.4%) and 117.5 (AUC, 0.798; 95% CI: 0.67, 0.93; sensitivity, 77.8%; specificity, 77.9%), respectively. Based on these cut-off values, patients were divided into two groups, and RFS was compared. Kaplan–Meier survival analyses indicated that patients with tumors with a higher SR (≥ 12.5) or AR (≥ 117.5) values at pre-NAC SE imaging had a significantly poorer RFS rate compared with those with a lower SR (<12.5) or AR (<117.5) values (both Log-rank P<.001). For the clinical-pathologic parameters, the Kaplan–Meier survival analysis demonstrated that a higher clinical T stage was significantly associated with worse RFS (Log-rank *P*<.001) (Fig. 5).

## Discussion

Due to the highly molecular and clinical heterogeneity of the breast cancer, it has differential response rates to NAC and varying RFS. With the increased utility of NAC, there is a need for more reliable tools for early prediction of pCR and RFS to aid treatment selection. Compared to conventional US, SE is a complementary technique that can provide additional information about tissue stiffness, which is associated with tumorigenesis and disease progression [9]. Our study findings highlight the value of SE in the early prediction of pCR and RFS in LABC patients treated with NAC. After 2 cycles of NAC, SR_2_ (OR, 1.50; *P* = .003) and ΔSR_2_ (OR, 0.01; *P* = .015) were independently associated with pCR. A higher clinical T stage (HR = 4.17; *P* = .005), and a higher SR (HR = 1.11; *P* = .002) and AR (HR = 1.06; *P*<.001) values at pre-NAC SE imaging were independently associated with poorer RFS. To our knowledge, this is the first study to evaluate the value of SE in predicting the RFS of LABC patients after NAC.

Several studies have investigated the value of sonoelastography in assessing or predicting treatment response to NAC in breast cancer, and the preliminary results were promising [11, 12, 23]. Hayashi et al. [23] demonstrated that the mean elastography scores categorized by the Tsukuba elasticity scoring system were significantly lower in the pCR group. A prior study reported that pre-NAC tumor stiffness measured by elastography has a statistically significant association with pathological response to NAC in breast cancer [24]. However, these studies only analyzed the pre-NAC SE findings of breast cancer. Our study further analyzed the pre- and during NAC SE findings, and found that both after one and 2 cycles of NAC, most of the SE parameters and their changes showed a significant difference between the pCR and nonpCR groups. In our study, there was no significant difference regarding pre-NAC SE parameters between the two groups. The reason for inconsistency may be related to differences in ultrasound elastography techniques, data analysis methods or patient selection.

To reduce unnecessary cytotoxic exposure, more attention should be paid to the ideal time-point for performing SE prediction during NAC. Our results demonstrated that the predictive performance of SE parameters after 2 cycles of NAC was significantly better than that after 1 cycle of NAC, and SR_2_ and ΔSR_2_ can be used as an early-response markers during NAC. Chemotherapy can induce alterations in tumor bio-mechanical properties like fibrosis and inflammation, thus leading to the decrease in tumor stiffness and a decline in SR. Fernandes et al. [25] reported that a significant difference in SR was observed between pCR and nonpCR groups as early as 2 weeks into NAC. In another recent study, SR demonstrated high sensitivity (97.7%) and moderate specificity (68.7%) for determining response even after the first cycle of NAC, and was proved to be the earliest predictor of treatment response in patients of LABC [13]. But these studies only assessed the role of SE in evaluating the response to NAC, and its value in predicting RFS was not covered.

Previous studies have demonstrated that breast cancer stiffness measured by elastography is significantly correlated with well-known poor prognostic factors such as lymph node metastasis, lymphovascular invasion and immunohistochemical biomarkers [26–28]. Breast cancers with more aggressive tumour phenotypes such as triple-negative and HER2-positive cancers tend to have higher stiffness values than those ER-positive cancers [29]. Evans et al. [30] demonstrated that preoperative stromal stiffness measured by SWE has independent prognostic significance for breast cancer-specific survival in invasive breast cancer patients. Higher maximum shear wave speed of the tumour on preoperative US was significantly associated with poorer disease-free survival in breast cancer patients [31]. Therefore, it is plausible that ultrasound elastography may be useful in predicting the RFS of breast cancer patients. However, to our knowledge, little is known regarding the relationship between pre-NAC SE imaging features and RFS in LABC patients after NAC.

Our results demonstrated for the first time that higher SR and AR values at pre-NAC SE imaging were independently associated with poorer RFS. Increased deposition and cross-linking of collagen which can enhance tissue stiffness, contributes to breast cancer formation and metastasis [32, 33]. A prior study by Acerbi et al. [34] demonstrated that the progression and transformation of breast cancer are accompanied by an incremental increase in collagen deposition. Tumour stiffness measured on SWE is demonstrated to be significantly correlated with tumor hypoxia [35]. The hypoxic tumor microenvironment is closely associated with angiogenesis, metastasis and resistance to treatment of the tumour [36]. In addition, a recent study by Alba et al. [37] reported that high tumour stiffness measured by SWE was strongly correlated with tumor growth and ECM crosslinking but negatively correlated with T cell migration, which may reduce the efficacy of anti-PD-1 therapy. Therefore, a higher SR value which indirectly reflects tumour stiffness, can reflect the potential of tumour progression and invasiveness and may be an early-prognostic maker of breast cancer survival.

AR was shown to be a reliable variable for differential diagnosis of breast tumors and the AR value of malignant lesions was statistically higher than that of benign lesions [36, 37]. High AR measurements were more frequently observed in breast cancer, especially in invasive breast cancer [38, 39]. Our study demonstrated that AR at SE was an independent pre-NAC predictor of RFS in LABC patients. A higher AR value may be an indirect manifestation of tumor invasive growth, and may have a higher probability of recurrence.

Our study had several limitations. First, conventional and SE US were performed by the same radiologists, hence the assessment of the SE features may be influenced by conventional US. Second, we have not evaluated inter- and intra-observer variability in SE imaging acquisition and interpretation. Third, the follow-up time varied in this study, ranging from 10 to 91 months. The follow-up period of 60.7 months was relatively short to evaluate late recurrence and overall survival. Finally, this was a retrospective interpretation of prospective data study, and the sample size was relatively small. But this study might reveal some underlying rules, and it may be helpful in guiding further prospective study.

## Conclusions

In conclusion, SR and its change were the best predictors of pCR after 2 cycles of NAC, and a higher SR and AR values at pre-NAC SE imaging were independently associated with poorer RFS. The results of our study suggest that SE can serve as a more clinically relevant approach to early predict pCR and RFS in LABC patients treated with NAC, and thus may offer valuable predictive information to aid personalized treatment selection. Further large-scale prospective research with longer follow-up period is needed to corroborate our findings.

## Data Availability

The datasets used and/or analysed during the current study are available from the corresponding author on reasonable request.

## Abbreviations

SE: Strain elastography
pCR: Pathologic complete response
RFS: Recurrence-free survival
NAC: Neoadjuvant chemotherapy
ES: Elasticity score
SR: Strain ratio
HP: Hardness percentage
AR: Area ratio
HR: Hazard ratio
ROC: Receiver operating characteristic curve
